# The Prognostic and Predictive Significance of circRNA CDR1as in Tumor Progression

**DOI:** 10.3389/fonc.2020.549982

**Published:** 2021-02-10

**Authors:** Fang Jian, Ren Yangyang, Xu Wei, Xu Jiadan, Li Na, Yang Peng, Bian Maohong, Niu Guoping, Pan Zhaoji

**Affiliations:** ^1^Department of Blood Transfusion, The First Affiliated Hospital of Anhui Medical University, Hefei, China; ^2^Clinical Laboratory, Xinyi People’s Hospital, Xuzhou, China; ^3^Department of Clinical Medicine, The First Affiliated Hospital of Anhui Medical University, Hefei, China; ^4^Clinical Laboratory, Xuzhou Central Hospital, The Affiliated XuZhou Hospital of Medical College of Southeast University, Xuzhou, China

**Keywords:** cerebellar degeneration-related protein 1 antisense, cancer, function, prognostic, predictive significance

## Abstract

Cerebellar degeneration-related protein 1 antisense (CDR1as) is an important member of the circRNAs family, also known as cirs-7. Its main function *in vivo* is to act as a mir-7 sponge. Accumulated studies show that CDR1as is closely related to various diseases, especially cancer. Our analysis show that CDR1as expression in human cancer is significantly associated with poor overall survival (hazard ratio [HR] = 2.50, 95% confidence interval [CI] = 2.06–3.04; *p* < 0.00001) and that high CDR1as expression is associated with the tumor node metastasis stage (odds ratio [OR] = 2.13, 95% CI = 1.63–2.78; *p* < 0.00001), and distant metastasis (OR = 3.50, 95% CI = 1.90–6.64; *p* < 0.00001). Furthermore, the results reveal the prognostic significance of CDR1as in neoplasms of the digestive system (HR = 1.69, 95% CI = 2.14–2.71; *p* < 0.001), colorectal cancer (HR = 1.34, 95% CI = 1.96–2.85; *p* < 0.001), and non-small cell lung cancer (HR = 2.40, 95% CI = 3.42–4.83; *p* = 0.008). In this study, we summarize in detail the latest research findings and demonstrate the function and regulatory mechanism of CDR1as in various cancer processes, and its potential as a biomarker for cancer prevention and prognosis.

## Introduction

In recent years, cancer has become the most serious threat to human health. There were more than 1.68 million new cancer patients in the United States in 2017, and more than 600,000 people died of cancer (1). Therefore, the prevention and treatment of cancer is one of the most urgent problems. Circular RNAs (circRNAs) are a class of RNA molecules that lack the 5’-3’ terminal and are covalently bound to form a closed loop and single-stranded transcripts from exons, introns, or intergenic regions (2, 3).

CircRNAs were first found in viroids in 1976, and were later isolated from several eukaryotes in 1979; they have been erroneously considered as transcriptional noise (4, 5). The role of circRNAs in disease regulation has not been adequately appreciated. However, with the rapid development of bioinformatics and high-throughput sequencing technology, the biological characteristics of circRNAs have been gradually revealed. Discovered in recent years, circRNAs are a large class of ncRNAs, widely found in a variety of eukaryotes, that participate in a variety of biological processes (6–8). Numerous studies have revealed the importance of circRNAs in regulating gene expression at both the transcriptional and post-transcriptional level. In addition, dyregulation of circRNA is regulated with the abnormal progression in various dieases, including cancer (9–14). To date, a variety of circRNAs have been shown to play a crucial role in human cancer initiation and development, including liver, lung, colorectal, breast, and prostate cancer (15). Sang et al. detected that circRNA_0025202 regulates tumor progression in breast cancer by regulating the *miR-182-5p/FOXO3a* axis (16). CircHIPK3 is significantly upregulated in colorectal cancer (CRC) tissues and cell lines and is an independent prognostic factor of poor overall survival (OS). Knockdown of circHIPK3 markedly inhibited CRC cell proliferation, migration, invasion, and induced apoptosis *in vitro* and suppressed CRC growth and metastasis *in vivo* (17). Liu et al. implied that bladder cancer patients with high circ0001361 expression levels have a poor OS and that circ0001361 promotes bladder cancer cell invasion and metastasis both *in vitro* and *in vivo* (18). Hsa_circ_0067934 is upregulated in esophageal squamous cell carcinoma (ESCC) tissues and may be a potential prognostic marker for ESCC (19). The upregulation of circRNA_100338 and downregulation of circMTO1, circ-ITCH, and cSMARCA5 are positively correlated with the poor prognosis of hepatocellular carcinoma (HCC) (20–23). It has been reported that Hsa_circ_0001649 has potential diagnostic and prognostic value for gastrointestinal tumors and may be a sensitive indicator for distant metastasis of gastric cancer and liver cancer (24–27). Thus, it is becoming increasingly clear that circRNAs are important in cancer pathogenesis. CircRNAs play important roles in the diagnosis, growth, metastasis, and treatment of drug resistance in cancer. Therefore, circRNAs are expected to be the new targets for tumor diagnosis and treatment (28, 29).

## Materials and Methods

### Literature Search Strategies

The public databases PubMed, EMBASE, Web of Science, Wiley Online Library, and Medline were searched with the terms “CDR1NAT” or “ciRS-7” or “CDR1as” and “Cancer” or “Tumor” or “Neoplasia” and “prognos*” or “surviv*” or “outcome” or “predict.” The last search was on December 1, 2020.

### Inclusion and Exclusion Criteria

All eligible study data were evaluated and extracted by two researchers; the relationship between CDR1as, tumor tissue, and patient survival was analyzed only when expression levels were reported. The inclusion criteria were as follows: (a) expression levels of CDR1as in cancer tissues; (b) correlation between the expression levels of CDR1as and survival outcomes (OS, progression-free survival [PFS], metastasis-free survival [MFS], or disease-free survival [DFS]); (c) hazard ratio (HR) and 95% confidence interval (CI) for survival time were reported or could be calculated from the reported data. Exclusion criteria were as follows: (a) Animal studies, case reports, meta-analysis articles, and reviews; (b) Papers lacking raw data or from which we could not calculate the HR, 95% CI, and *p* values.

### Data Extraction

We extracted the following data: first author’s name, publication year, study region, sample size, cancer type, tumor size, tumor node metastasis (TNM) stage, the method of CDR1as testing, outcome, HR with 95% CI, and HR statistics from each eligible study.

### Statistical Analysis

STATA 12.0 (StataCorp, College Station, TX, USA) was used in this study. HR, OR, and corresponding 95% CIs were used as indicators to evaluate each clinical parameter and patient prognosis or survival. *p* < 0.05 was considered statistically significant.

## Results

### Biogenesis of CDR1as

Cerebellar degeneration-related protein 1 antisense RNA (CDR1as), also known as asciRS-7, is a circRNA that is transcribed in the antisense orientation with respect to the *CDR1* gene (30, 31). CDR1as is predominantly found in the human brain, it is 1485bp in length and located on the X chromosome (chrX: 139865339–139866824) (32). It functions as a miR-7 sponge/inhibitor in the embryonic zebra fish (33), and it is also expressed in a variety of tissues and organs in the human body. Its expression imbalance is associated with a variety of diseases, such as Alzheimer’s disease, diabetes, myocardial infarction, and cancer (34–36). CDR1as has diverse biological functions and it is mainly involved in the proliferation, migration, and invasion of tumor cells. It regulates the biological function of tumors in two main ways (1): binding to the miRNA as a competing endogenous RNA through the base complementary pairing principle (2); regulating gene expression at the transcriptional and post-transcriptional level (2, 13, 15, 37). Numerous studies have shown that CDR1as is dysregulated in many human malignancies, such as CRC, non-small cell lung cancer (NSCLC), HCC, and gastric cancer (GC); they may serve as new diagnostic biomarkers and cancer treatment targets (38, 39). We systematically reviewed the literature to provide information on CDR1as expression patterns and roles in various tumors.

### The Multifaceted Role of CDR1as in Human Tumors CDR1as in Hepatocellular Carcinoma and Cholangiocarcinoma

**Figure 1:**
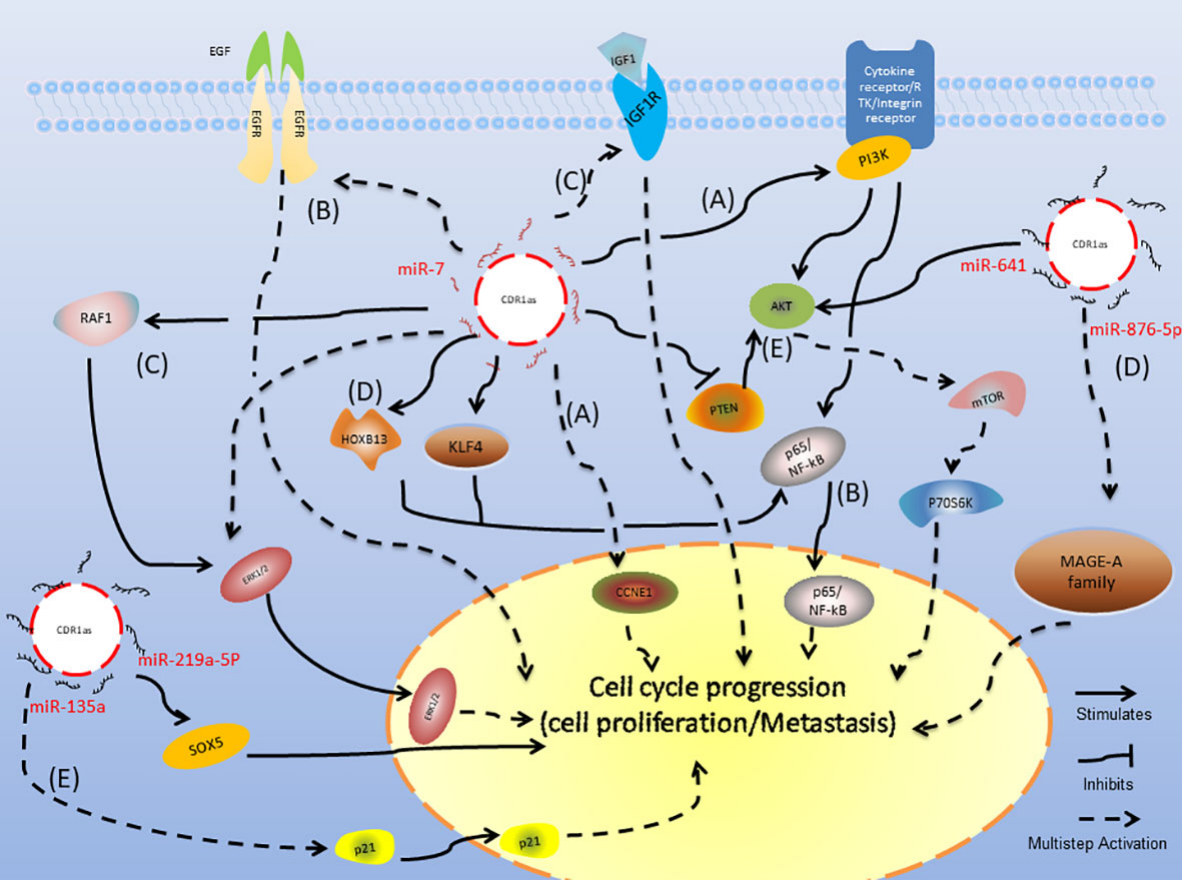
Mechanism of CDR1as in various cancers. **(A–C)** CDR1as regulates the signaling pathways of HCC, NSCLC, and CRC processes. **(D)** CDR1as regulates the signaling pathways of ESCC processes. **(E)** CDR1as regulates the signaling pathways of other cancers.

Recently, four research groups have analyzed the expression of CDR1as in HCC and its relationship with prognosis (40–43) (**Table 1**). Although the expression of CDR1as in HCC tissues remains controversial, upregulation of CDR1as is an independent risk factor for hepatic microvascular invasion (MVI) and promotes the proliferation and invasion of HCC cells by inhibiting the expression of mir-7 and its downstream target genes *CCNE1*, *PIK3CD*, *KLF4*, and *p70S6K* (**Figure 1A**). Jiang et al. evaluated the relationship between CDR1as expression levels and clinicopathological features of cholangiocarcinoma (CCA) and assessed the association of CDR1as expression with OS. The results showed that upregulation **of** CDR1as was closely associated with advanced TNM stage, lymph node invasion, and postoperative recurrence, and the OS of patients with CCA with high CDR1as expression was worse than that of patients with CCA with low CDR1as expression (48). CDR1as binds to Mir-641 and accelerates the degradation of Mir-641, which may lead to upregulation of relative mRNA levels of *AKT3* and *mTOR* and thus promote tumor progression (49). These data indicate that CDR1as plays an important regulatory role in the progression of HCC and may provide insights for the treatment of HCC and CCA.

**Table 1:** Summary of the role of CDR1as in cancers so far.

Cancer type	Function	Expression level	Intersection molecules and/or pathway	References
NSCLC	Promoting cell proliferation, migration and invasion	Up	CDR1as-miR-7/NF-κB or CDR1as-miR-7-EGFR/CCNE1/PI3K pathway; CDR1as-miR-219a-5p/SOX5 pathway	(44–47)
HCC	Promoting hepatocellular microvascular invasion	Up	CDR1as-miR-7-EGFR or PI3K/AKT/mTOR pathway; CDR1as-miR-7-EGFR/CCNE1/PI3K pathway; CDR1as-miR-7-KLF4 pathway	(40–43)
CCA	Promoting patients Lymph node invasion and correlated with advanced TNM stage	Up	CDR1as-miR-641/AKT/mTOR pathway	(48, 49)
CRC	Promoting cell proliferation, invasion and correlates with advanced tumor stage, tumor depth and metastasis	Up	CDR1as-miR-7-EGFR/IGF-1R; CDR1as-miR-7-EGFR/RAF1/MAPK pathway	(50, 51)
ESCC	Promoting cell proliferation, migration and invasion	Up	CDR1as-miR-7-HOXB13/NF-κB; CDR1as-miR-876-5p-MAGE-A family pathway; CDR1as-miR-7/KLF4/NF-κB pathway	(52–54)
Brain tumor	Inhibiting cell proliferation and migration	Down	CDR1as-miR-671-5p-VSNL1 pathway; CDR1as-p53/MDM2 complex pathway	(55, 56)
Head and neck cancer	Promoting cell proliferation, migration and invasion and correlates with TNM stages, poorly differentiated tumours, lymph node metastases and poor prognosis	Up	CDR1as-miR-7-CCNE1/PI3K pathway; CDR1as-miR-7-5p-E2F3 pathway	(57, 58)
Melanoma	Promotes invasion *in vitro* and metastasis *in vivo*	Down	CDR1as-IGF2BP3 axis pathway	(59, 60)
Bladder cancer	Inhibiting cell proliferation, invasion and migration; induced the apoptosis and enhanced the cisplatin chemosensitivity	Down	CDR1as-miR-135a-p21 pathway; CDR1as-miR-1270/APAF1 axis pathway	(61, 62)
GC	An independent risk factor of overall survival; Correlates with a more aggressive oncogenic phenotype	Up	CDR1as-miR-7-PTEN/PI3K/AKT pathway	(63)
Osteosarcoma	Impairing cell vitality and increased apoptosis and G1/S arrest in parallel with reduced ability of cell migration	Up	CDR1as-miR-7-EGFR/CCNE1/PI3K/RAF1pathway	(64)

HCC hepatocellular carcinoma CCA, cholangiocarcinoma; NSCLC, non-small cell lung cancer; CRC, colorectal cancer; ESCC, esophageal squamous cell carcinoma; GC, gastric cancer.

### CDR1as in Non-Small Cell Lung Cancer

In non-small cell lung cancer tissues, the expression of CDR1as is upregulated compared with para-carcinoma tissues. The dysregulation of CDR1as is significantly related to the clinical features and prognosis of NSCLC (44). Su et al. (45) confirmed that upregulation of CDR1as could inhibit the expression of *miR-7* and that it correlates with advanced histopathological grade, larger tumor size, and more severe lymph node metastasis. Furthermore, researchers have confirmed that overexpression of CDR1as and downregulation of *mir-7* increases the proliferation, metastasis, and invasion ability of NSCLC cells by the CDR1as/*miR-7*/*NF-kB* (*p65*) axis (**Figure 1B**). Zhang et al. demonstrated that patients with high expression of CDR1as had a high TNM stage, more frequent lymph node metastasis, shorter OS time, and that CDR1as was an independent prognostic factor for patients with NSCLC. Mechanistically, CDR1as functioned as a miR-7 sponge to upregulate the key target genes of *miR-7*, including *EGFR*, *CCNE1*, and *PIK3CD* (46) (**Figures 1A, B**). Li et al. indicated that knockdown of circCDR1as inhibited the progression of NSCLC by decreasing cell viability, migration, and invasion, and increasing apoptosis by upregulating *miR-219a-5p* and downregulating *SOX5* (47). These studies indicate that CDR1as may play an important role in the development of NSCLC and may contribute to the diagnosis and prognosis of NSCLC.

### CDR1as in Colorectal Cancer

Tang et al. found that the expression of CDR1as was upregulated in CRC tissues compared to the adjacent normal mucosa. High expression of CDR1as was positively correlated with tumor size, T stage, lymph node metastasis, and poor OS. Dysregulation of CDR1as inhibited CRC cell proliferation and suppressed *EGFR* and *IGF-1R* expression by blocking miR-7expression (50) (**Figures 1B, C**). Weng et al. analyzed the clinical significance of CDR1as in CRC patients and found that CDR1as was significantly upregulated in CRC tissues compared with matched normal mucosae. Furthermore, they revealed by multivariate survival analysis that CDR1as could be an independent risk factor for OS. Upregulation of CDR1as led to blocking of *miR-7* and resulted in a more aggressive oncogenic phenotype, and subsequent activation of *EGFR* and *RAF1* oncogenes in HCT116 and HT29 cells (51) (**Figure 1C**). These findings suggest that CDR1as could be a prognostic biomarker in CRC patients and may serve as a therapeutic target in CRC patients.

### CDR1as in Esophageal Squamous Cell Carcinoma

A study performed by Sang et al. suggested that CDR1as is upregulated in ESCC and that it is associated with poor clinicopathological parameters in ESCC patients. High expression of ciRS-7 increased the proliferation, migration, and invasion abilities of ESCC cells. Mechanistic studies revealed that CDR1as acts as a sponge for miR-876-5p in ESCC cells. Furthermore, CDR1as reverses miR-876-5p-mediated repression of the tumor antigen MAGE-A family in ESCC cells (52) (**Figure 1D**). Li et al. (53) evaluated the expression of CDR1as in 123 ESCC patients and found that CDR1as expression was significantly upregulated in ESCC tissues compared with normal tissues. High expression of CDR1as was positively correlated with poor survival and advanced TNM stage, and inhibited the tumor inhibition of miR-7, including cell proliferation, migration, and invasion. Research on the mechanisms revealed that CDR1as functioned as a sponge for miR-7 and reactivated its downstream HOXB13-mediated *NF-κB* pathway (**Figure 1D**). Huang et al. demonstrated that the expression of ciRS-7 was significantly increased in ESCC tissues and cells compared with their corresponding controls, and that ciRS-7 triggers the migration and invasion of ESCC *via* miR-7/KLF4 and NF-κB signals (54). These studies indicate that CDR1as may play an important role in the development of ESCC and might contribute to the diagnosis and prognosis of ESCC.

### CDR1as in Brain Tumor

CDR1as was identified as a circRNA in the brain and it is highly expressed in normal brain tissues (65). Barbagallo et al. observed that the expression of CDR1as is significantly decreased in glioblastoma multiforme biopsies and compared to normal brain parenchyma (average change= −3.51-fold), in glioblastoma multiforme cell lines and other cancer cell lines, except HCT116. Furthermore, the researchers identified CDR1as as a downstream miR-671-5p target in glioblastoma multiforme and that it is the only circRNA known to be targeted and degraded by miR-671-5p (55, 65). Lou et al. revealed that CDR1as expression decreased with the increase in glioma grade and that it was a reliable independent predictor of overall glioma survival. In terms of mechanism, CDR1as functions as a tumor suppressor by binding directly to *p53* at its DBD region to restrict MDM2 interaction (56).

### CDR1as in Head and Neck Cancer

Zhang et al. collected 30 laryngeal squamous cell carcinoma (LSCC) tissues and corresponding relative normal tissues to determine the expression of CDR1as and their clinical significance. The results indicated that patients with high TNM stages, poorly differentiated tumors, lymph node metastases, and poor prognosis had high CDR1as levels. Overexpression of CDR1as enhanced cell vitality and promoted the proliferation, migration, and invasion of two LSCC cell lines. Mechanistically, CDR1as functioned as miR-7 sponges and upregulated the key targets of *miR*-7, *CCNE1*, and *PIK3CD* (57). Zhong et al. examined 44 nasopharyngeal carcinoma (NPC) tissues and 20 non-tumor tissues, and the results confirmed that CDR1as was highly expressed in NPC tissues and cell lines, which might be related to the promotion of NPC cells development. Moreover, CDR1as could upregulate *E2F3* expression by binding to miR-7-5p, and promote the growth and glucose metabolism of NPC cells (58). These results indicate that CDR1as is an oncogene in NPC and LSCC.

### CDR1as in Melanoma

Zhang et al. predicted the regulatory role of CDR1as in melanoma through bioinformatics analysis and showed that CDR1as may act as a competing endogenous RNA for the vital genes, which are associated with the invasion and migration of melanoma (59). Recent research has revealed that epigenetic silencing of *LINC00632* can lead to the depletion of CDR1as and promote invasion *in vitro*, and metastasis *in vivo* through a *miR-7*-independent, IGF2BP3-mediated mechanism (60). These results indicate that CDR1as plays an important regulatory role in the biological process of melanoma.

### CDR1as in Bladder Cancer

Li et al. found that the expression of CDR1as in bladder cancer was significantly lower than that in adjacent tissues. CDR1as overexpression inhibits the proliferation, invasion, and migration of bladder cancer cells and directly binds to miR-135a and inhibits its activity in bladder cancer (61) (**Figure 1E**). Yuan et al. reported that Cdr1as induced apoptosis and enhanced the chemosensitivity of bladder cancer cells both *in vitro* and *in vivo*. We verified that CDR1as exerts a cisplatin-chemosensitization effect on bladder cancer cells through the CDR1as/*miR-1270*/*APAF1* axis (62).

### CDR1as in Other Cancers

A study by Pan et al. investigated the clinical significance of CDR1as in 102 primary gastric cancer tissues and matched para-carcinoma tissues, and subsequently confirmed the clinical relevance in an independent validation cohort. The results indicated that CDR1as expression was higher in gastric cancer tissues than in para-carcinoma tissues, and increased with the tumor stage. The overexpression of CDR1as blocks tumor inhibition induced by miR-7 in MGC-803 and HGC-27 cells by antagonizing the miR-7-mediated *PTEN*/*PI3K*/*AKT* pathway (**Figure 1E**), leading to a more aggressive oncogenic phenotype (63).

Uhr et al. revealed the relationship between miR-7 and its binding compound CDR1as and the prognosis and prediction of first-line tamoxifen treatment for breast cancer (66). Xu et al. showed that CDR1as was upregulated in osteosarcoma tissues and its expression was positively correlated with tumor size and distant metastasis. Knockdown of CDR1as leads to de-repression of miR-7 levels and inhibition of its target genes, including *EGFR*, *CCNE1*, *PI3KCD*, and *RAF1*, impaired cell vitality and increased apoptosis and G1/S arrest in parallel with reduced cell migration ability (64). In addition, CDR1as has been reported to play an important role in the development of ovarian cancer and pancreatic cancer (67, 68).

### CDR1as as a Biomarker in Cancer

**Figure 2:**
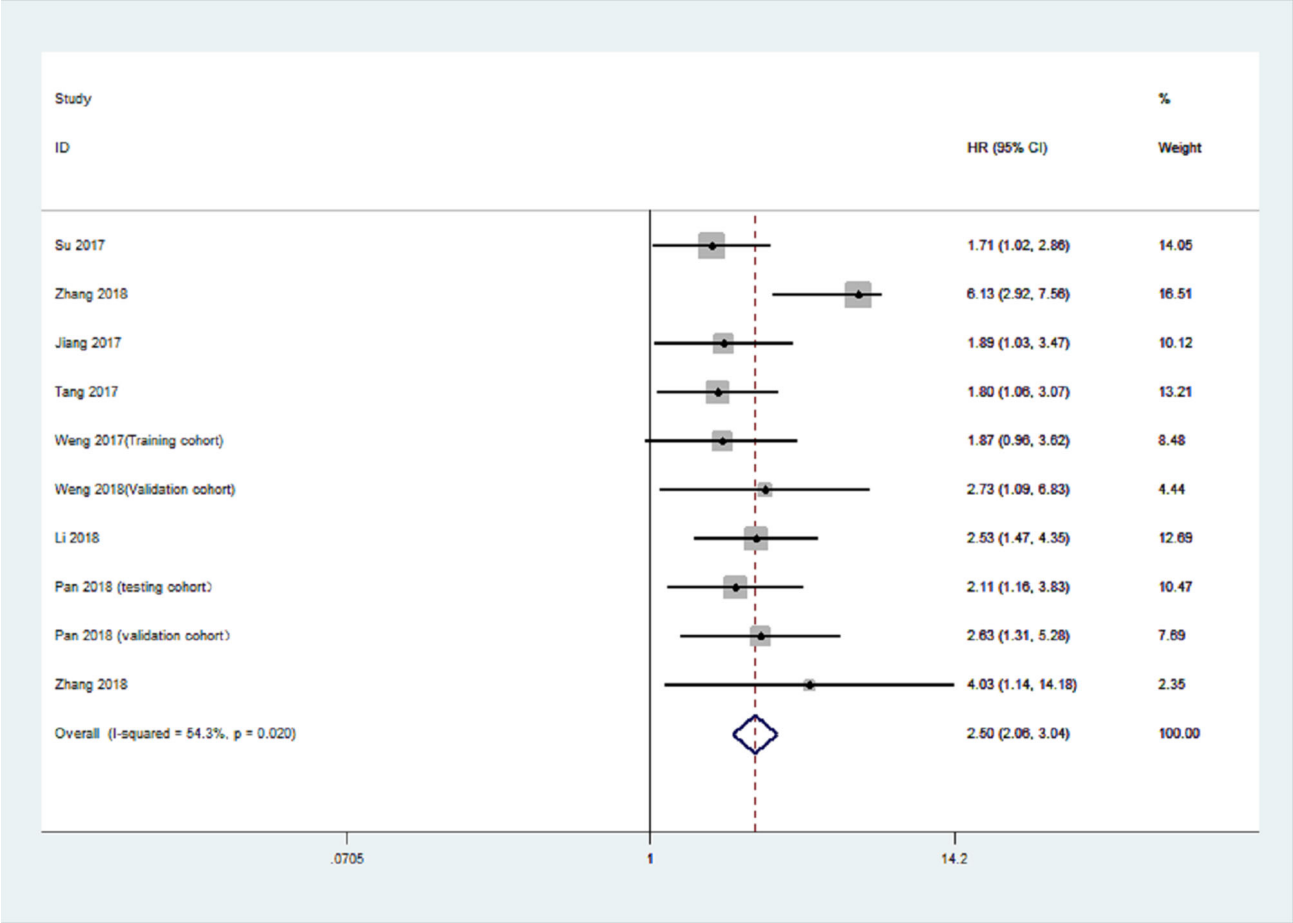
Forest plot of the hazard ratio (HR) for CDR1as increase and overall survival.

**Figure 3:**
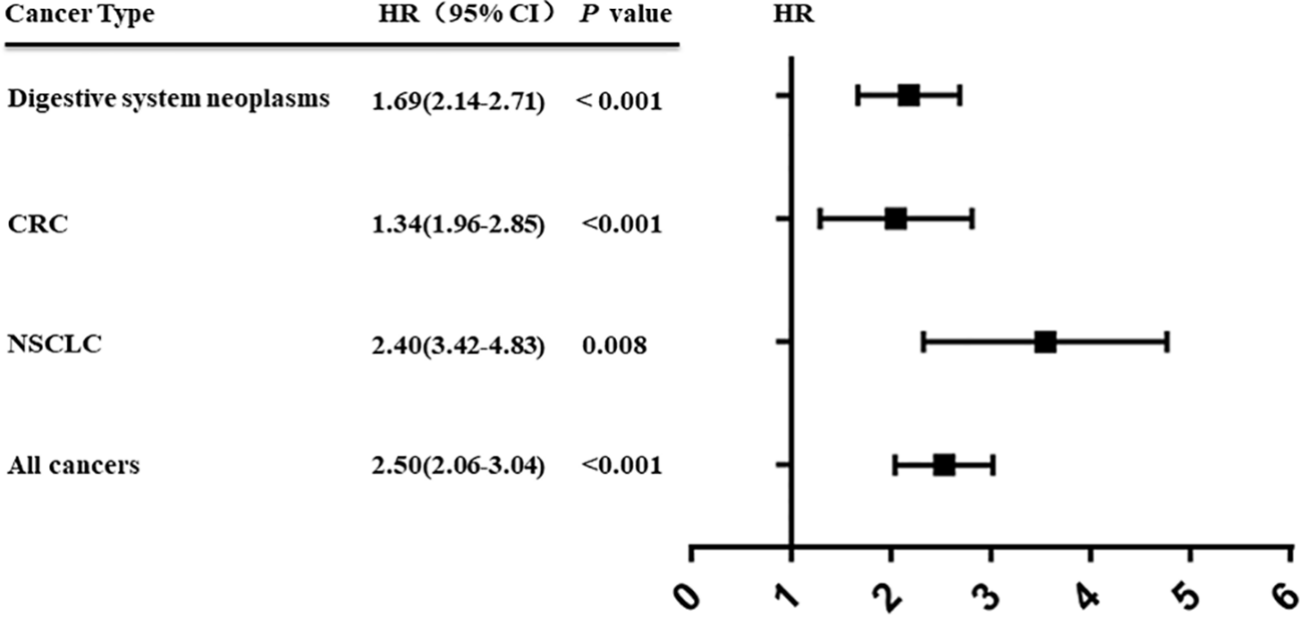
High expression of CDR1as indicates poor prognosis of tumor.

**Figure 4:**
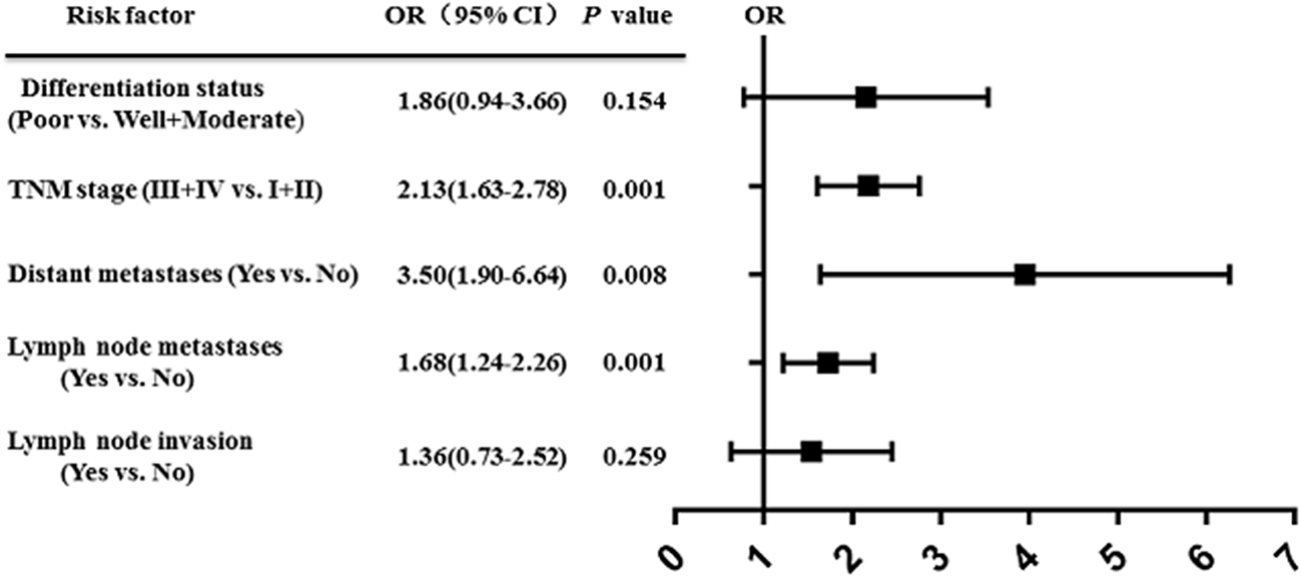
High expression of CDR1as is related to the clinicopathology of tumor.

An increasing number of studies have shown that upregulation of CDR1as is associated with prognosis in various types of cancer (69, 70). We performed a comprehensive literature search from a public database (PubMed, Medline, and Web of Science) to identify all eligible studies describing the use of CDR1as as a prognostic factor for different types of cancer. A total of eight studies were included in this study (45, 46, 48, 50, 51, 53, 57, 63). The results showed that high expression of CDR1as was significantly associated with poor OS (HR = 2.50, 95% CI: 2.06−3.04; *p* < 0.001) (**Figure 2**). Furthermore, the results revealed the prognostic significance of CDR1as in digestive system neoplasms (HR = 1.69, 95% CI = 2.14−2.71; *p* < 0.001), CRC (HR = 1.34, 95% CI = 1.96−2.85; *p* < 0.001), and NSCLC (HR = 2.40, 95% CI = 3.42−4.83; *p* = 0.008) (**Figure 3**). High expression levels of CDR1as were also associated with TNM stage (OR = 2.13, 95% CI = 1.63–2.78; *p* = 0.001), distant metastases (OR = 3.50, 95% CI = 1.90–6.64; *p* = 0.008), and lymph node metastases (OR = 1.68, 95% CI = 1.24−2.26; *p* = 0.001), but not with the differentiation status and lymph node invasion (**Figure 4**). These results suggest that the expression of CDR1as may be an effective biomarker for the diagnosis, treatment, and prognosis of tumors.

## Conclusions

At present, circRNA has become a new field in disease research, including diseases such as autoimmune (71), gynecological (72, 73), respiratory system (74), and cancer (75–78). CircRNAs not only play an important role in tumor regulation, but can also be used as biological markers for tumor diagnosis and treatment. It has been pointed out that Hsa_ circ_0000190 has a low expression level in gastric cancer plasma and tissues, and its sensitivity and specificity as a diagnostic marker are even better than CEA and CA19-9 (79). Hsa_circ_0005075 has high specificity and sensitivity for the diagnosis and treatment of HCC (80). Therefore, the expression and characteristics of circRNAs can be used as biological markers of tumors.

As a well-known circRNA, CDR1as plays an important role in the progression of many diseases. In recent years, more studies on the pathogenesis, progression, and mechanism of CDR1as have been conducted. Differences in the expression of CDR1as in various tumors are obvious. CDR1as regulates the biological behavior of tumor cells in many aspects, including cell proliferation, differentiation, metastasis, and apoptosis. CDR1as mainly regulates gene expression at the transcriptional and post-transcriptional levels. It is a strictly controlled biological process, rather than a random splicing error. Its main form of action in cancer is as a miRNA sponge, which makes the competing endogenous RNA network more complex. Furthermore, we integrated the prognostic information of CDR1as in different tumors, and found that the higher the expression of CDR1as, the shorter the survival time, and the worse the prognosis, especially in patients with digestive system tumors. In addition, the evaluation of TNM stage, distant metastasis, and lymph node metastasis in tumor patients are of great value.

In this study, we reviewed the characteristics, biological functions, and clinical application value of CDR1as in detail, and mainly examined its expression in various tumors, its influence on prognosis of tumor patients, and its role in tumor signaling pathways. The high expression of CDR1as in tumors promotes the ability of tumor cells to differentiate, proliferate and metastasize, leading to the poor prognosis of cancer patients. Although the current research on CDR1as is not very thorough, and its detailed mechanism for regulating tumor progression is not very clear, CDR1as plays an irreplaceable role in the process of tumorigenesis and may become a potential target for cancer prevention and treatment.

## Data Availability Statement

The datasets presented in this study can be found in online repositories. The names of the repository/repositories and accession number(s);, can be found in the article/supplementary material.

## Author Contributions

All authors have contributed to the preparation of this manuscript. FJ, RY, XW, and XJ were responsible for collecting the data, analysis, and drafting the first copy. YP and LN were responsible for editing the manuscript. PZ, NG, and BM were responsible for the theme, final editing, and preparation of the manuscript for submission. All authors contributed to the article and approved the submitted version.

## Funding

This study was supported by the Natural Science Foundation of Anhui Province (grant number 1808085 MH273) and the first Affiliated Hospital of Anhui Medical University National Natural Science Foundation Cultivation fund (grant number 2018kj24).

## Conflict of Interest

The authors declare that the research was conducted in the absence of any commercial or financial relationships that could be construed as a potential conflict of interest.
